# Supplementary material for: LC-AMP-F1 Derived from the Venom of the Wolf Spider Lycosa coelestis, Exhibits Antimicrobial and Antibiofilm Activities
Source: Pharmaceutics. 2024 Jan 19;16(1):129. doi: 10.3390/pharmaceutics16010129 (PMC10818355; doi:10.3390/pharmaceutics16010129)
Supplement: Supplementary file 1 [file pharmaceutics-16-00129-s001.zip › pharmaceutics-2764629-supplementary.pdf]

Supplementary Information

LC-AMP-F1 derived from the venom of the wolf spider *Lycosa coelestis*, exhibits antimicrobial and antibiofilm activities compared with Melittin

Yuxin Song [1], Junyao Wang [1], Xi Liu [1], Shengwei Yu [1], Xing Tang [2,*] and Huaxin Tan [1,*]

1. The Key Laboratory of Ecological Environment and Critical Human Diseases Prevention of Hunan Province Department of Education, Department of Biochemistry and Molecular Biology, School of Basic Medicine, Hengyang Medical School, University of South China, Hengyang, Hunan, 421001, China
2. Hunan Key Laboratory for Conservation and Utilization of Biological Resources in the Nanyue Mountainous Region, College of Life Sciences, Hengyang Normal University, Hengyang 421002, China

Email: huaxintan@usc.edu.cn; xtang2018@hynu.edu.cn

Table S1. Physical stability of LC-AMP-F1 against *E. coli*. The change of MIC under different temperature (**a**) and pH (**b**).

a

**Temperature**	**MIC (μM)**	
	LC-AMP-F1	Melittin
37 °C	5	5
80 °C	5	5
100 °C	5	5

b

**pH**	**MIC (μM)**	
	LC-AMP-F1	Melittin
5	10	2.5
6	10	5
7	5	5
8	5	5
9	10	5

Table S2. Salt sensitivity of LC-AMP-F1 in *E. coli*.

**Peptide**	**MIC (μM)**	
	LC-AMP-F1	Melittin
**Control**	5	5
**150 mM NaCl**	20	10
**4.5 mM KCl**	10	5
**6 μM NH_4Cl**	5	5
**1 mM $MgCl_2$**	20	10
**2.5 mM $CaCl_2$**	>80	20

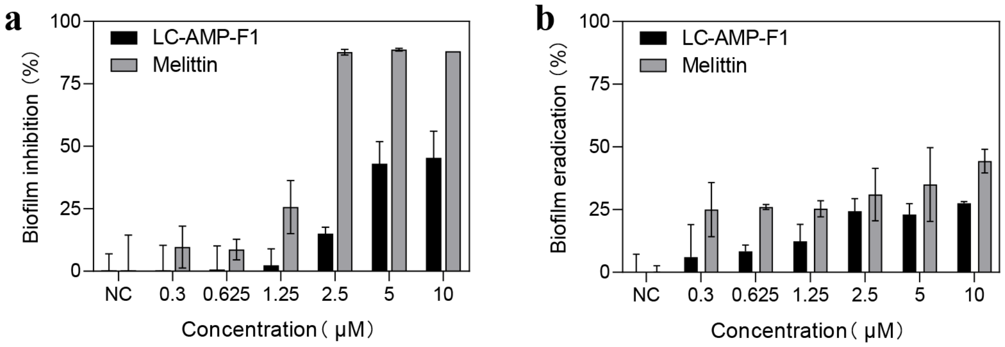

Figure S1. Inhibition (a) and eradication (b) activities of LC-AMP-F1 on 1065 biofilm.
